# Insights into the challenges and facilitators to physical activity among brooklyn teens enroled in a weight management programme

**DOI:** 10.1111/hex.13528

**Published:** 2022-05-27

**Authors:** Viola R. Browne, Denise M. Bruno, Sarita Dhuper, Aimee Afable

**Affiliations:** ^1^ School of Public Health State University of New York (SUNY) Downstate Health Sciences University Brooklyn New York USA; ^2^ Department of Pediatrics State University of New York (SUNY) Downstate Health Sciences University Brooklyn New York USA

**Keywords:** childhood obesity, health disparities, real‐world evaluation, urban health

## Abstract

**Purpose:**

A qualitative study was carried out to explore obese adolescents' understanding of physical activity, perceptions of the ideal body type and to identify facilitators of and barriers to physical activity.

**Methods:**

Twenty‐two adolescents 12–18 years of age and 14 of their parents were recruited from an obesity intervention programme in Brooklyn, New York, from June to November 2017. Data were collected using focus groups and individual semi‐structured interviews, followed by interpretative phenomenological analysis of the transcripts.

**Results:**

The adolescents wanted to ‘lose some weight’, but not to be ‘thin’ or ‘look hungry’. Most females desired a ‘slim‐thick’ figure, which was ‘a flat stomach with big thighs, and curvy’. Fun and support from parents, peers and programme staff facilitated achieving their physical activity goals. Barriers included low self‐efficacy, inactive families, fear of neighbourhood gangs and crime and perceptions that the parks were small and overcrowded, with limited physical activity options for adolescents.

**Conclusion:**

These findings highlight the need to consider local norms concerning body image when designing obesity interventions. To effectively reduce childhood obesity in New York City, policy should prioritize the promotion of public safety, improvement of neighbourhood parks and increase options for physical activity.

**Patient or Public Contribution:**

The voices and narratives of patients and their families informed this study.

## 1 BACKGROUND

In 2017–2018, the prevalence of obesity among US youth 2–19 years of age was 19.3%, and 21.2% of adolescents 12–19 years of age were obese.^1^ There are also racial and ethnic disparities in obesity, with increased prevalence among non‐Hispanic Blacks (29.1%), and Hispanic (23%) adolescents when compared with their White (14.8%) and Asian (5.1%) peers.^1^ Obesity is also more prevalent among socioeconomically disadvantaged groups.^2^, ^3^ Central Brooklyn is one of the poorest areas in New York City (NYC). Most residents are Black and experience a disproportionate burden of obesity‐related diseases.^4^, ^5^ Obesity rates in children 5–14 years of age are among the highest in NYC at 23%–25%.^6^

Childhood obesity has been accompanied by an increased prevalence of hypertension and Type 2 diabetes, other obesity‐related comorbidities^7^, ^8^ and a lower health‐related quality of life.^9^ Childhood obesity persists into adulthood,^10^ and is associated with higher early adult mortality.^11^ Effective obesity interventions are needed to improve health outcomes and reduce the high medical costs of treatment of children with excess weight and obesity‐related conditions.

A combination of physical activity, reduced sedentary behaviour and dietary modification is recommended for the reduction of obesity and related comorbidities.^12^, ^13^ However, the Youth Risk Behaviour Survey found that most adolescents do not meet the physical activity guidelines. There are also disparities in physical activity levels, with black and Hispanic adolescents less active than their white peers.^14^

Decreasing adolescent obesity is a public health challenge. Adolescence is a critical period of development,^15^ where lifestyle and health‐related behaviours are established and the risk for obesity is increased.^16^, ^17^ Overweight and obese adolescents are less active than normal‐weight adolescents.^18^, ^19^ They face additional challenges to engage in physical activity, are victimized by peers and experience higher rates of low self‐esteem and loneliness than their peers who are normal weight.^20^

Previous studies indicate that intrapersonal, interpersonal and community factors affect obese adolescents' physical activity behaviours.^21^, ^22^, ^23^ Facilitators include motivation to improve their health^24^, ^25^ and appearance,^26^ and family and peer support.^25^, ^27^ The availability of physical resources such as parks and recreation facilities in their neighbourhoods, and opportunities to be physically active in school have also been reported as facilitators.^23^, ^28^

Barriers to physical activity have included lack of motivation,^26^, ^28^, ^29^ lack of knowledge and misperceptions of physical activity,^30^ cultural attitudes about weight^29^, ^31^ and misperceptions about their weight.^31^, ^32^ Heightened body consciousness, including insecurity about their appearance, avoidance of humiliation, bullying and lack of social support,^20^, ^33^ concerns about neighbourhood safety,^28^ lack of resources and limited access to community resources^31^, ^34^ have also been reported as barriers to physical activity.

A systematic review of qualitative studies by Stankov et al.^35^ identified common barriers to physical activity faced by adolescents who were overweight or obese including negative body and unsafe neighbourhoods. However, although socioeconomic status was not adequately considered, the review points to potential variation by sex, race/ethnic group and socioeconomic status. For example, there seemed to be greater acceptance of obesity reported in studies among African‐American girls, relative to studies in White and more affluent populations.^35^ Additionally, there are few recent qualitative studies of the perceptions related to physical activity among adolescents with obesity who reside in low‐income inner‐city neighbourhoods.

The Social‐Ecological Model postulates that multiple levels of influence (intrapersonal factors, interpersonal processes and primary groups, institutional and community factors and public policy) affect behaviour, and that the different levels interact with each other.^36^ It is a framework to examine physical activity.^37^ The exploration of the lived experiences of adolescents with obesity from communities of colour, their perceptions of the intrapersonal, social and environmental facilitators and barriers to being physically active may improve understanding. This is important for development of sustainable intervention programmes. The purpose of this qualitative study was to explore the facilitators and barriers for physical activity among adolescents with obesity who reside in Central Brooklyn, New York, and enroled in a tertiary care obesity intervention programme. The objectives were to explore the concepts involved in the perception of physical activity among adolescents with obesity, their perceptions of the ideal body type and the personal, social and environmental factors that facilitate and present barriers to physical activity.

## 2 METHODS

### 2.1 Setting

Participants were recruited from the *Live Light Live Right (LLLR)* tertiary care childhood obesity programme.^38^ *LLLR* utilizes a multidisciplinary approach (medical evaluation, nutrition education, physical activity and behavioural counselling)^38^ and is the only community‐engaged obesity treatment programme serving children in Brooklyn.

Institutional Review Board approval for the study and all study material was received from SUNY Downstate Health Sciences University. Participants in the study were recruited from June to November 2017.

### 2.2 Recruitment and sample

Using purposeful sampling, we recruited adolescents 12–18 years of age with overweight/obesity, who were English speaking, enroled in the *LLLR* programme. We also recruited one parent of each child for interview. We distributed separate recruitment flyers to the adolescents and their parents during their medical appointments or during their attendance at exercise sessions, and they were invited to participate. We explained the general purpose of the study to potential parents/adolescents who expressed interest, and those who agreed to participate were guided through the assent and consent procedure. Recruitment procedures yielded 33 adolescents aged 12–18 years and 27 interested parents.

### 2.3 Data collection

A female Caribbean American doctoral‐level researcher (V. B.), who is a registered nurse with maternal child health experience, led this study.^39^ Her interest in the health and development of children of colour living in disadvantaged neighbourhoods was the motivation for this study. She volunteered at *LLLR* and exercised alongside the children, conscious of her position as an ‘insider’ because of similar racial and socioeconomic backgrounds with many of the participants but also as an ‘outsider’ to these adolescents and their families because of her position as a female researcher affiliated with an academic institution, and her professional experience. She was aware of her preconceptions and acknowledged that the researcher's biases may have influenced the data collection and analysis.

Separate focus group and semi‐structured interview guides were prepared for the adolescents and parents. The interview guides are included in the Supporting Information Appendix. The adolescent interview guides (Appendix S1) focused on perceptions of physical activity, body image and factors that affect physical activity. Parent guides (Appendix S2) were similar, but did not include adolescents' body image. Four focus groups and 14 individual semi‐structured interviews were scheduled and conducted in private rooms at two exercise sites with 22 adolescents and 14 parents living in the Central Brooklyn area from August to November 2017. We conducted two same‐gender focus group interviews with adolescents, ages 12–15 years, that consisted of seven female and six male adolescents. The literature indicates that there are gender differences in adolescents' physical activity levels,^40^ and in their attitudes towards physical activity.^41^ Stratification by gender may have elicited more in‐depth information.

We also conducted two focus group interviews with five mothers of 12–15‐year‐old female adolescents, and four mothers of 12–15‐year‐old male adolescents. When the focus group participants chose their seats, their initials were written on a seating chart for note‐taking of significant comments or reactions. The interviews were audiotaped and were 45–60 min in duration. Due to low turnout for the focus groups with older adolescents (ages 16–18 years) and their parents, we offered them the option of individual semi‐structured interviews. We interviewed 14 participants, 9 older adolescents (16–18 years) and 5 parents who expressed agreement at exercise and clinic sites, with each interview lasting 25–30 min. Each adolescent and parent were given a $20 and $40 gift card as an honorarium.

### 2.4 Analysis

Data collection and analysis occurred simultaneously. Interpretative phenomenological analysis (IPA) was the approach used as the guiding perspective to explore the lived experiences of the adolescents and their parents in their social and community environments.^42^ IPA aims to explore in detail how participants make sense of their personal and social world, to examine the meanings that particular experiences and events hold for participants and to deepen or extend understanding of the participants' experiences.^43^, ^44^

The audio tapes were transcribed verbatim and analysed as soon as possible following the focus group and individual interviews. Notes were taken to document ideas, and the researcher's conceptual thoughts and reflections as they occurred during the analyses. Each transcript was read several times and distinctive phrases and emotional responses were highlighted. We identified participants' statements by gender and their age groups (younger adolescents: 12–15 years, older adolescents: 16–18 years) to respect their request that we do not use pseudonyms in the report.

Each highlighted item was assigned a code and organized into emergent themes that were relevant to the aim of the study. The themes were grouped according to conceptual similarities, re‐examined for significance and assigned descriptive labels. Themes from all of the adolescent interviews were combined into a list to reflect the findings. This process was repeated with the themes from the adult interviews. In the final phase, these lists were combined in a list of superordinate themes and organized to reflect participants' perceptions. A journal with memos and notes was utilized in conjunction with the interview. During this process, the researcher used ‘reflexivity’ for awareness of personal biases, assumptions and beliefs that she brought to the study^38^ and deliberately ‘bracketed’ them.^45^ Data collection and analysis continued until after four focus groups and 14 individual interviews, when we reached thematic saturation and no new themes emerged.

### 2.5 Validity considerations

We evaluated this study utilizing rigour as recommended by Lincoln and Guba.^46^ Prolonged engagement to understand the lived experiences of the adolescents and families enhanced credibility. The researcher volunteered at the programme sites and exercised with the adolescents and families to become familiar with them to build trust and rapport. Rigorous techniques and methods (audio recording and note taking) garnered high‐quality data from multiple sources about the adolescents (focus groups, individual interviews and notes). Prolonged engagement with the data provided in‐depth information of the adolescents' physical activity perceptions and behaviours.^43^, ^46^ Member‐checking with participants confirmed that the summary of findings that we shared with them reflected their experiences and perceptions,^46^ and helped to validate the conclusions.^47^ Detailed information is provided about the participants, the composition of focus groups and the contexts and assumptions underlying the study. The researcher's actions, changes and the rationale for them, notes and reflections were recorded in a journal throughout the study. Another member of the research team evaluated whether the findings, interpretations and conclusions were supported by the data.

## 3 RESULTS

The adolescents had a mean age of 14.9 years, were 50% male and the majority were African‐America/Black (86%). Twenty‐one participants were obese/severely obese (body mass index [BMI] percentile >95th), and one participant was overweight (BMI percentile 90th), and the average BMI was 36.6 (range: 25.3–57.5). Sixteen adolescents had been attending the obesity clinic or enroled in the exercise programme for more than 6 months and six for less than 6 months. Among the partent participants the majority were female (13/15) and were African‐American/Black (13/15).

The main themes that emerged from the participant interviews are presented here, organized according to Socioecological Model (SEM) levels.

### 3.1 Individual and interpersonal factors

#### 3.1.1 Adolescents' body image: ‘slim‐thick’ and ‘toned and neat’

All the adolescents wanted to lose weight; 19 adolescents expressed the desire to change their appearance. However, adolescents revealed that they did not embrace a thin body as their ideal and this was not their motivation for physical activity.

‘To be at the right body size’, ‘To be fit’, and ‘I want to you know, not skinny, skinny, but lose some weight’.

Gender differences emerged about how the ideal body type was constructed. Most male adolescents wanted to look slim ‘toned’ and muscular, but did not want to ‘look hungry’.

According to an older male, ‘I do it because also I want to look more toned instead of looking like wide and I would say sloppy. I'd like to look more toned and neat’.

**Figure 1:**
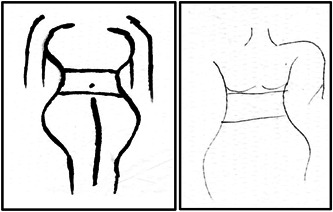
Younger female adolescents' drawings of the ‘slim‐thick’ image.

Conversely, females desired the ideal body type that was ‘slim‐thick’. When probed, a younger female described this as ‘when you are skinny, and you got a big butt, your stomach is flat, but you still got curves’. Two younger female participants volunteered to draw the image to demonstrate exactly what they meant. Figure 1 shows younger female adolescents' drawings of the ‘slim‐thick image’.

An older female articulated the body type and changes desired as, ‘I do not like my arms; I feel that they are huge. My legs are too big. My stomach is, I just feel like my entire body is disgusting. I want my stomach to go down, and I want like the sides to be toned, and I want my legs to be smaller’.

Television and music industry celebrities were a common ideal. Younger female adolescents admired the bodies of females of hip hop artists or reality television stars such as Nikki Minaj, Kim Kardashian, Tyra Banks and Amber Rose. Two older females reported admiration for the shapes of a female plus‐size model, and the ‘slim‐thick’ figure of a female exercise trainer in their exercise programme. The male adolescents admired men with muscular bodies such as a star on ‘Iron Chef’, Gordon Ramsey, and basketball players such as LeBron James, Stephen Curry and James Harden.

The adolescents also had grounded expectations about their body ideals, the difficulty in attaining them and understood that some celebrities' shapes were acquired artificially.

I am not going to go all the way. Some people do plastic surgery to look like that. We don't want the plastic surgery part. (Younger female)

They perceived that hard work and exercise were more realistic approaches to weight loss, improving their muscle tone and appearance and attaining one's ideal, as revealed in the following statements:

If I exercise and I get toned, then it's just like something that happens. (Older male)

You not going to get like that because if you're working out. The trainer will make you exercise your arms and thighs as well. So, you gonna lose the weight in your arms and thighs and everywhere. (Younger female)

Yeah, my favorite basketball player is LeBron James and I believe he works for all the stuff he does. He just inspires me to work to my expectations. (Older male)

This older female's statement about the plus‐sized model revealed that her reality was grounded in her acceptance of her size:

She embraces what she has. She has insecurities with herself. Like you could relate to her. She has insecurities about her love handles so she shows it off more, ‘like I have insecurities I'm not perfect.’ So, I like her for that.

#### 3.1.2 Gender differences in motivations for physical activity

Gender differences emerged in physical activity behaviours. Most male participants enjoyed playing team sports with their peers in their neighbourhood or school and having friendly competitions.

Playing basketball with my friends that was easy ‘cause it wasn't like a serious atmosphere’. It was like jokes and stuff, so I didn't feel pressured to do well. You know I could mess around, and I was still getting exercise. (Older male)

Yeah I'm competitive. When I was racing, I came with full energy. I kinda wanted to run but I was upset he was tired. I actually wanted to see how fast he was. (Younger male)

Few female participants reported that they enjoyed team sports, and most preferred dancing and playing fun games. They did not want to exercise or play sports or compete with peers. They felt that they could exercise when their performance was not being judged:

When it was easy for me to exercise, I was alone. I was happy, and I kept pushing myself. I kept doing what I had to do. I felt happy because in that moment I felt like I was doing it for me. I didn't think about how my mom would see me, how my dad would see me, how others, my friends, nobody. I didn't care how they would see me. I cared about how I would see myself. (Older female)

While both male and female adolescents expressed similar specific exercise goals, including weight loss to improve their appearance for school events, and to wear more flattering clothing, there were differences. Male participants engaged in repetitive exercise/workout for muscle building. Conversely, female participants expressed dislike for repetitive activities, and that they did not want to be muscular or slim.

I feel like I'm really working, I'm working for something, I'm earning it because even though it's hard for me, I know that I am going to succeed or something good is going to happen to me. You keep in mind that even if you don't like doing it in the long run it will benefit you, this would help. You always have to keep in mind that you're doing it for a good reason and you're helping yourself. (Older male)

Some exercise can also turn your fat into muscle which is not what I want (younger female). Table 1 presents exemplary quotes about the gender differences in motivation for physical activity.

**Table 1:** Statements about gender differences in motivation for physical activity.

Gender	Exemplary statements
Female	‘I consider volleyball for physical activity because it's fun’. (Older female)
	‘Working out and going to the gym, I just don't like working out like push‐ups and running, I don't like it’. (Older female)
	‘Some things you can have fun with like swimming, jumping’. (Younger female)
	‘Before summer I was graduating from middle school. So my goal was, you got prom, you got graduation and that's what you're here for. So yeah I look good in my prom dress, and now I'm fat again’. (Younger female)
Male	‘I saw him race first and then him race (points to two other boys). They were like really fast, so that also makes me want to push myself to be faster’. (Younger male)
	‘When I was going against this dude, he beat me twice, but it made me, like after the second time it made me want to push myself a little more. So, I like competition because it makes me want to do more’. (Younger male)
	‘I try to keep up because I kinda walk around with extra weight. So, keeping up is something that I had to do to, like you know to be, I guess to look cool in other people's eyes’. (Older male)
	‘I wanted to get picked, and like the other kids they want you to do it again, especially in basketball when you get picked. I want to get picked again. So, I always wanted to be able to keep up, so I had a higher chance of being picked’. (Older male)

#### 3.1.3 Low self‐esteem and fear of ridicule

Low self‐efficacy emerged as a barrier for physical activity when the adolescents described their feelings about the difficulties that they experienced with activities. Parents also revealed that the adolescents' perception of their weight affected their self‐confidence.

I just have no confidence to do it. I feel that exercising is important, but I feel like I just have no self‐confidence, not only to do it in school, but like just going to a gym or going to a program. (Older female)

I think when it comes to exercising, they get discouraged. (Mother of younger female)

The adolescents' negative self‐perceptions and the fear of being ridiculed by their peers, and at school and in their neighbourhoods affected adolescents' self‐esteem and self‐efficacy, and discouraged physical activity:

Certain friends should not be there around when you are exercising. Some people will just be there on the sidelines, looking on the sidelines laughing. (Younger female)

In my neighborhood if they see a person like me, a big person run or try to walk, sometimes the adults could, not all of them, but some people just come by, just walk by. They would make comments. Or like the very hood guys would say something and laugh. (Older female)

### 3.2 Group and community factors

#### 3.2.1 Facilitators and opportunities for physical activities

In addition to the expectation that physical activity should be fun and engaging, the adolescents felt that their parents provided tangible support by taking them to exercise sessions, and emotional support when they encouraged them to go out and to exercise. Some parents held similar perceptions about how their support helped their children.

My dad he's really, ‐ I won't exercise unless he says something. Like he literally has to drag me to the field to do some walking. (Younger male)

Sometimes he doesn't want to, but I will push him and say let's go, we're going for a walk we're going to the park. The entire summer after work I'll say meet me by the subway, bring your bike, I'll walk, and you'll ride. (Mother of younger male)

Supportive settings and social networks in and out of school also encouraged physical activities.

One friend that would push me a lot, he will always be with me to make me go the furthest that I can go, and always encourage me. So, that helps a lot. (Older male)

They were all supportive when we were in gym, like rooting for me if I was playing softball. (Older female)

My gym teacher noticed, he pushed me to get on the court and play basketball and that's when I became more active running around and being active. He believed in me, he would always push me to my extent, to the fullest I could go because he knew I can do it. (Older male's description of his experience in a new school)

Support from trainers of the *LLLR* programme was also instrumental as the following statements reveal:

The reason why we laugh, and joke is because I feel the trainers, even though it's painful or hard, they always try to put a smile on our faces and never give up. They motivate us to just do it, and do it, and do it. (Older female)

I'm looking forward to him seeing there's other kids that's going through the struggles. So, he doesn't feel like he is alone doing it by himself and that I'm forcing it on him. This is another venue for him to have support from kids his age. (Mother of younger male)

Table 2 presents additional exemplary statements and insights of the participants' perceptions of facilitators.

**Table 2:** Exemplary statements about facilitators to physical activity.

Theme	Exemplary statements
Fun	‘It's fun, I think it's more fun exercising at school than in the real world. Because you have classmates you could just do it together, and it's not really exercising. It's like team sports and it's building instead of like exercising’. (Older female)
	‘Some things you can have fun with like swimming, jumping’. (Younger female)
Social support	‘My mother, my father, my sister my brother. they're always telling me, they're always pushing me to go outside and do something, go outside and run or something. I feel like that's really helping because if I wanted to do it by myself, I don't think I would continue. They give me the mindset to always make sure you're always keeping up to do what you have to do’. (Older male)
	‘I have friends that were supporting me. I was on the basketball team, and everyone wanted me to play. Being in a comfortable environment, when you have friends and then the other people on the other side, I find it balances out and then I just go’. (Older male)
	‘He is over, ‐ obese. I try to make him do some exercise, not to sit around, move around a little. Keep his blood flowing, his heart pumping the right way. So, I think it's very important to me and to him. I am okay with him doing physical activity. I push him because sometimes he lies back. So, I always push him and say “you need to do a little exercise even in the house. You get up for fifteen, twenty minutes, do something instead of sitting around”. I try to make him active. I even made him do swimming, just to keep him active’. (Mother of older male)
*Live Light Live Right*	‘I like how she pushes me. Even when she is being mean, like you know that deep down is not because it's like she is “I hate you”. It is because “I want to see you succeed”’. (Younger female)
	‘…different stuff you all try to incorporate into it. The basketball program, the step thing, the dancing, the cardio stuff, it gives kids the option if you don't want to work out. Not everybody works out the same. People want to do different things. So, you created more options for everyone, that's good’. (18‐year‐old male)
	‘At *Live Light Live Right* everybody is in the same boat. So, it makes it easier to work out and balance out with everyone. Seeing other people improve over time from all the work out, it motivates you to also improve. So, it's like a fun competitive atmosphere’. (Older male)
	‘The program will make it easier for physical activity; coming here’. (Parent of older male)

#### 3.2.2 Barriers for physical activity

Similar to adolescents' fear of ridicule, parents had similar beliefs of the potential negative effects on their children:

My son was telling us he was uncomfortable going outside because there were some kids that were saying things about him. Some kids form the neighborhood that were making comments about his weight and making him uncomfortable. I guess he felt, ‘if I don't go outside, they're not gonna bother me’. (Mother of younger male)

#### 3.2.3 Family constraints: Lack of support, family behaviour

The adolescents' perceptions about the effect of family constraints included lack of support, sedentary families and parental inconsistencies as barriers to exercising or engaging in physical activity. Some adolescents reported that they had no support, and their parents acknowledged that they were not supportive. Inactive or inconsistent behaviours among their family members were also perceived as negative influences on the adolescents' behaviours:

Right now, I have none really. There's always people that say they want me to change but it's not much of pushing me to do something, my mom, some cousins, friends. My dad more of them wanting me to be in sports to use myself for football…. No not much support from them. (Older male)

I don't give them much support. ….When you come home, and you have a full day of work, I mean, I'm sleepy, I am tired, I am overworked. I leave work late every day now. I am getting up early I want to come home and literally go to sleep. I gotta make sure they eat’. (Mother of two younger males)

#### 3.2.4 Neighbourhood constraints

Negative perceptions of the neighbourhood were barriers to the adolescents' physical activity. They depicted the neighbourhoods as unsafe with crime, the fear of being bullied and the avoidance of gangs as reasons to avoid going outdoors.

But the one thing I am afraid of is somebody getting killed again. Because somebody got killed on my block. (Younger female)

A unanimous sentiment among the younger males was that ‘there are bullies out there’. An older male described how this affected him:

People like gang members and stuff are that in the area. I try to stay away from that stuff or around them.

Parents' perceptions about neighbourhood safety emerged via concerns about “supervision of our children” and “no guarantee they'll be safe when they go out there,” and their unwillingness to allow their children to go outdoors.

…but going to the park, no, I don't think it's safe, I don't think is safe. He is a boy, so no, I don't want anybody to be recruiting him to be a gang member. No!. (Mother of younger male)

The adolescents depicted the neighbourhood and parks as overcrowded and small, with limited equipment for physical activity as barriers. Parents also expressed their frustrations about the limitations of the neighbourhood parks and the lack of facilities such as community centres and after school programmes.

In my neighborhood that's really it because we have a packed neighborhood. There is not really like open area where you could go through houses or go around the block with, like, open spaces. It's just packed apartment buildings together. (Older female)

In my neighborhood there is just nothing really, it's just the park, the Betsy Park. That's it there's nothing for him… So basically, there is no facility there for us. (Mother of younger male)

**Figure 2:**
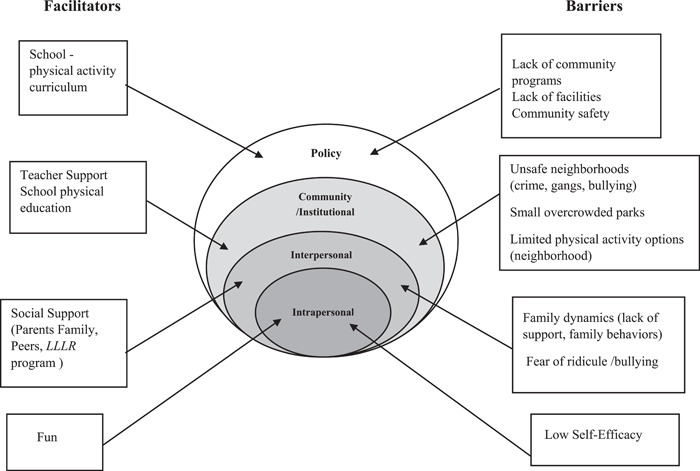
Adolescents' perceived facilitators and barriers for physical activity.

Table 3 presents additional exemplary statements about barriers to physical activity. Additionally, Figure 2 shows the participants' perceptions of facilitators and barriers in the context of the SEM.^37^, ^38^

**Table 3:** Exemplary statements about barriers to physical activity

Theme	Exemplary statements
Low self‐esteem, fear of ridicule	‘In my mind there's always doubts; that I wouldn't be able to complete something, and sometimes that stops me from wanting to exercise. Sometimes it's overwhelming’. (Older male)
	‘So, like when I work out with other people around me, I get so non‐confident because everybody around me is mad skinny, bony, like anorexic. Then I am walking looking like this. I'm like, I can't jump because of these two things (holds her breasts) going to jump with me. I can't do anything because these people, they flat chested, and me with my big old self going and jumping. That can't happen!’. (Younger female)
	‘The one thing I don't like which is the relay races when you have to go against, because they don't put you in your weight class. You're going against some skinny person that's been working out since three years old, haven't been eating nothing. I'm halfway behind or fall. I don't do that. I don't do relay races’. (Younger female)
	‘Having friends that are different size from you and smaller than you, sometimes you want to run around. You wonder why they run faster than you. They're able to do a lot of stuff that you can't do. So, it's overwhelming. Maybe there'll be a few of us running or jogging somewhere, and you know, only been able to go a quarter of the trac before stopping, it hurts, make me not have confidence in myself’. (Older male)
	‘In school there are a lot of slim teenagers, especially in the classes I have. I am like the biggest, so it's just uncomfortable. Sometimes it could be embarrassing, and then sometimes I don't push myself as I would because I really don't want to push myself in front of people. They make it so, not about me, but if they see a big kid doing something, they would either make a comment about them or something. Even if it's joking, it'll still be like awkward, the staring, the whispering behind my back’. (Older female)
	‘The one thing I don't like which is the relay races when you have to go against, because they don't put you in your weight class. You're going against some skinny person that's been working out since three years old, haven't been eating nothing. I'm halfway behind or fall. I don't do that. I don't do relay races’. (Younger female)
	‘I think he is a little depressed I think with the weight and stuff like that. I guess if he loses some of the weight, he would have more energy and he would push himself more’. (Mother of older male)
Family constraints—lack of support family behaviours	‘My whole family is heavy except for a few skinny ones. They let their weight get out of control. They exercise when they feel like it. Lazy, they just tired, tired’. (Older female)
	‘My aunts, all of them on that side they do not exercise. All they want to do is go out and eat all the time. No none of them. They're all fat too, for no reason’. (Younger male)

	‘My mom and dad sometimes they say we going to exercise, and we can't do it every day, ‐ either they don't want to do it’. (Younger male)
	‘I have no motivation to exercise’ and ‘exercising is not on my mind right now’. (Mother of younger female)
	‘I have a brother with a disability so a lot of times I just stay home to help. So, I never really get a chance to go out. Sometimes I'd be exhausted. I also have a little brother, 2‐ years‐old that I usually watch and take care of a lot. Being with them just makes you want to stay inside a lot. I may stay and not do what I want to do, like go outside and run around’. (Older male)
Neighbourhood constraints (lack of safety and limited options)	‘You get jumped in my park, every time you look at people’. (Younger female)
	‘But she wouldn't go and walk. She doesn't like going to the park by herself. She doesn't trust going to the park by herself. For safety reasons. Just for safety reasons’. (Mother of older female)

	‘I'm a very antisocial person and I am very, very protective of my daughter. … I live in the projects. I'm not gonna let her walk to the park. This is my baby and there (sic) is too many nasty men outside. So, I am very scared about that. I am not letting her out of my eyesight by herself’. (Mother of younger female)
	‘It's just not as good as the others. It's just that they are small. Prospect Park and Marine Park they have the trees and stuff. The parks that I like are big parks and these are smaller. I guess in places like Marine Park you could go to the park and other places like restaurants or something that you could just hang out at. But these they don't really have those. In other neighborhoods its more things you can do and more things you'd want to do. But ours you just go home and go to work and school’. (Older male)
	‘He says it's not safe it's a ghetto. From his point of view, he thinks its ghetto, so he doesn't go outside’. (Mother of older male)
	‘The program, that's the only one that I know about’. (Mother of older female)

## 4 DISCUSSION

This qualitative study aimed to explore the perception of physical activity among adolescents with obesity, their perceptions of the ideal body type and the personal, social and environmental factors that facilitate and present barriers to physical activity.

The finding that the adolescents did not perceive a thin image as their ideal body type is contrary to a study of African American girls aged 11–14 years, who viewed a slender frame as an attractive body size.^48^ This is consistent with a previous study of African American adolescent girls who did not embrace a slim image and were satisfied with their bodies, but expressed specific areas for change. However, in that study, the BMI status of those participants was unknown.^49^ The male adolescents' desire to be toned is consistent with the study of obese adolescents in the Bronx, New York, in which many male participants desired developed muscle mass and less body mass.^24^ Although the previous studies revealed that the adolescents were comfortable with their larger body image, the current finding of the specific ‘slim‐thick’ figure is novel and is a characterization of the ideal body type that resonated with Brooklyn female adolescents we interviewed. Local and cultural norms concerning body image should be a consideration in planning intervention programmes.

The influence of media personalities differs from previous studies with African American girls. In several studies, participants compared their bodies and appearances to images in the media and embraced the slim images of models.^48^, ^49^ It has been suggested that as the number of African American women in the media increases, African American girls may begin to use these images for social comparison.^50^ The participants' internalization of the celebrity body types as ideal can potentially place them at risk for engaging in unhealthy weight loss behaviours to attain the desired shapes.

The scepticism of the reality of the body types portrayed in the media is consistent with prior research on African American adolescent girls who recognized that some celebrity attributes were fake^48^ and unrealistic, and the strategies used by media and celebrities to hide flaws and change.^49^ Despite the idealized ‘thick‐thin’ image, these adolescents' media literacy may be a protective factor for engaging in healthy weight‐related behaviours.

Gender differences in the norms and motivations for physical activity among the adolescents are consistent with previous studies of adolescents with obesity.^23^, ^24^ Consideration of these differences in motivation and exercise preferences and providing female adolescents with options for noncompetitive activities can potentially improve their physical activity behaviours.

Low self‐esteem and perceived inabilities prevented adolescents from participating in physical activity in various settings. Prior research supports the importance of self‐efficacy for exercise among adolescents.^51^, ^52^ According to Bandura, people tend to avoid situations they believe exceed their coping skills and participate in those they judge that they are capable of doing.^53^

Heightened body consciousness (insecurities about their appearance, the fear of ridicule and bullying), perceived as a barrier by these adolescents, is similar to previous qualitative studies.^20^, ^33^ Salvy et al.^54^ indicated that experiencing negative peer influences appeared to deter youth from being physically active, and that children and adolescents may avoid physical activities in an attempt to avoid further weight criticism, teasing and victimization from peers in general.

The perception of family dynamics as barriers is consistent with prior research that found that the lack of parental motivation prevented obese African American adolescents from being physically active.^26^ These findings highlight the importance of social support and family behaviours for the inner‐city adolescents with obesity.

The negative perceptions of the parks, safety concerns and the fear of gangs as barriers to physical activity is consistent with previous studies that generally report about the fear of unsafe neighbourhoods.^28^, ^30^, ^34^ They highlight the need for neighbourhood improvements in low‐income inner‐city communities.

### 4.1 Policy and practice implications

This study provided insights about adolescents with obesity in a low‐income neighbourhood that has not been explored previously. The use of the SEM highlighted the need to combat childhood obesity at multiple levels of influence to promote and sustain active lifestyles into adulthood. At the individual level, obesity interventions need to consider local norms concerning body image, reduce stigma and support realistic goal setting when engaging in physical activity. At the group or community level, strategies need to consider how teens model their behaviour after their family members and seek support from their peers as well as older adult mentors in schools (gym teachers) or in other supervised group settings (organized sports). At the policy level, there is an urgent need for NYC to adopt a multisectoral approach to promote public safety and increase options for physical activity. Policy changes are needed to improve the built environment, neighbourhood safety to address gang activity and crime and park maintenance. Community programmes and facilities to increase recreational options are required to better meet the needs of adolescents in low‐income neighbourhoods.

### 4.2 Strengths and limitations

The use of focus groups and individual interviews in this study elicited rich data and insights about how the adolescents with obesity from low socioeconomic and disadvantaged neighbourhoods perceive the cultural and contextual facilitators and barriers to physical activity. It also filled the gap in knowledge about these inner‐city adolescents' perceptions of their ideal body type, and the influences on their perceptions. However, this is a small purposeful sample of adolescents with obesity and their parents who live in traditionally socially disadvantaged and medically underserved neighbourhoods. Participants were recruited from an obesity intervention programme, and they also volunteered to participate in the study. Their perceptions may be different from those of other adolescents. Therefore, the findings may not be generalizable to the broader community.

## 5 CONCLUSION

This study found that the factors that affect these obese adolescents' physical activity are complex and interrelated and are important considerations for public health education and incorporation in obesity interventions. The adolescents' perceptions of ideal body types highlight the need to consider local norms related to body image/type when designing obesity interventions. To effectively reduce childhood obesity in NYC, policy should centre on the promotion of public safety, improvement of the built environment and neighbourhood parks and increasing options for adolescents' physical activity.

## CONFLICT OF INTEREST

The authors declare no conflict of interest.

## Supporting information

Supplementary information.

## Data Availability

The data that support the findings of this study are available on request from the corresponding author. The data are not publicly available due to privacy or ethical restrictions.
