# Supplementary material for: Insights into the challenges and facilitators to physical activity among brooklyn teens enroled in a weight management programme
Source: Health Expect. 2022 May 27;25(4):1832–43. doi: 10.1111/hex.13528 (PMC9327863; doi:10.1111/hex.13528)
Supplement: Supplementary file 1 — Supplementary information. [file HEX-25--s001.docx]

**APPENDIX 1**

**Moderator Guide for Focus Groups with Adolescents**

Introductions

Introduce self and give statement of purpose:

“We are here today to talk informally and get your ideas about physical activity and the things that affect your activity”

Introduce observer and ask permission to record.

“Please write your name or whatever you would like to be called today.”

“Great, now let us take a few minutes to introduce yourself; and perhaps say what you what you like to do in your spare time or after school.”

Wonderful, now let’s get started with our discussion.

Personal

1. How do you feel about exercising?

- Can you share how you feel when you are exercising?

- What happens when you are active?

2. What do you think about when you are going to be physically active/exercise?

- And while you are exercising?

-What makes you want to exercise?

- Are you exercising to look a particular way?

- Can you tell me about people that you admire and want to look like?

3. I would like you to share what you know about physical activity

- What are some of the good things about physical activity?

- What about things that you don’t like?

Interpersonal

4. Who are the people you exercise with?

- How do you feel when you are with them?

5. Who are the people that support you to be physically active?

- Your parents, - how do they affect your exercise?

- What are somethings that help you?

- Some things that prevent you from exercising?

- Other family members?

- Your friends?

Community/Organization

6. What is it like to be physically active in school?

- What things at school promote physical activity?

- What things make it difficult?

- What is it like in gym class?

- Rules?

7. What is it like exercising in the program?

- What makes you want to be in it?

- What do you like about it?

- What do you dislike about it?

8. Where can you go in your neighborhood to exercise?

- What is it like in your neighborhood for young people to be physically active?

- What about your neighborhood helps?

- What makes it hard to exercise in your neighborhood? How do you feel about that?

General

8. I would like you to share some of your experiences of when it was easy for you to exercise?

- What about when it was difficult?

9. What ideas do you have about what would make it easy for you to exercise?

10. Is there anything you would like to share?

- Anything I forgot to ask?

Closing

Thank participants

Debriefing as needed

Give gift card in an envelope

**APPENDIX 2**

**Moderator Guide for Focus Groups with Parents**

Introduction

“We are here today to talk and get some ideas about adolescents and physical activity, and some of the things that affect them.”

Introduce observer and ask permission to record.

Please write your name or whatever you would like to be called today.

Great, now let us take a few minutes to introduce ourselves, and perhaps you can say something you like to do.”

Wonderful, now we can get started with our discussion.

General Questions

1. How do you feel about exercising?

- Please share how you feel when you are physical active?

- How do you feel about your adolescent doing physical exercise?

2. What is it like when your children are exercising?

- What are some of the things they say that they feel?

3. How active would you saw your children are?

-How so?

4. Who provide support to your adolescent so he/she can be physically active?

- What things about your family help adolescents to be active?

- What makes it difficult for them?

5. What things in your adolescents’ schools help them to be physically active?

- What prevents physical activity?

6. Where can young people go in the neighborhood to exercise or play sports?

- What in your neighborhood makes it easy for young people to be physically active?

- What makes it difficult?

7. Can you please share what is good about your adolescents being physically active?

-What makes it hard?

8. What ideas do you have about what would make it easier for your adolescents to be physically active?

Is there anything you would like to share?

Anything you believe I forgot to ask?

Closing

Thank participants

Debrief as needed.

Give participants gift card in envelope

**Guide for Individual Interview with Adolescents**

Introduction:

This interview is being done to try and understand what it is like for adolescents to exercise and be active. The information you share will help to better understand affect adolescents’ physical activity. The interview will be audio taped. Your name will not be used in the final report.

1.What does physical activity mean to you?

- Is it the same as exercise?

- how important is physical activity to you?

-To your health?

- How much physical activity do you think adolescents should get?

- How do you feel about exercising?

- What do you think about when you are going to exercise?

- What does it feel like when you are exercising?

2.What is the reason that you exercise?

-How does physical activity affect the way you look?

- Are you comfortable with the way you look?

- Do you exercise because you want to change how your body looks?

- Please tell me a little more about that?

- What about people in the television and music industry who you admire?

- Are there any that you want to look like?

- Tell me more…

3. Who are the people you exercise with?

- How do you feel when you are with them?

4. Is physical activity common among your family members?

-Who are the people that support you to be physically active?

- In what ways do they help you to be active?

-Parents? –Other family members? Your Friends? - Other people?

- in what ways do they make it hard for you to be active?

5. What is it like trying to be active and exercising in school?

- In gym class?

-Recess?

-What things in school make it easy for you to be active? -What makes it hard?

6. Tel me about your neighborhood; What is it like for you to be active in your neighborhood?

- Where can you go in your neighborhood to exercise?

- What is it like in your neighborhood for young people to be physically active?

- Where can young people can go?

- How do you feel about that? -Are there other programs other than Live Light Live Right that help you to be physically active?

7. What is it like exercising in the program?

- What makes you want to be in it?

- What do you like about it?

- What do you dislike about it?

8. I would like you to share some of your experiences of when it was easy for you to exercise?

- What about when it was difficult?

9. What ideas do you have about what would make it easy for you to exercise?

10. Is there anything you would like to share?

Thank participant and give Gift Card

**Guide for Individual Interviews with Adults**

Introduction: Explain the purpose of the interview; “I will like to talk to you about adolescents and their physical activity and your ideas about what affects them. We will talk about your child and adolescents in general.”

1. What does it mean to live healthy?

- What does physical activity mean to you?

- Is it the same thing as exercise?

- How important is physical activity to you?

- To your health?

- To your child’s health?

- How do you feel about exercising?

- How much physical activity should adolescents get?

- How do you feel about your adolescent doing physical exercise?

2. What is it like when your child is exercising?

-- What are some of the things he/she says that he/she feel?

3. How active would you say your child is?

-How so?

4. Tell me about your role in your family;

- Do you help members of your family to exercise? How?

- Is physical activity common among your family members?

- In what ways do you think your child’s family and friends encourage him/her to be more active?

- In what ways do family members make it hard?

- What about your child’s friends; in your opinion are they physically active?

5. In your opinion, what things in your adolescents’ schools help him/her to be physically active?

- What makes it hard?

6. How do you feel about your neighborhood?

-Tell me about your neighborhood;

-Is it easy for your adolescent to be physically active in your neighborhood?

- What makes it easy?

- What makes it hard?

- Where can young people go in the neighborhood to exercise or play sports? - Are there any other groups or programs besides the Live Light Live Right Program that promote physical activity for adolescents in your neighborhood?

7. Can you please share what is good about your adolescent being physically active?

8. What ideas do you have about what would make it easier for your adolescent to be physically active?

Conclusion:

Is there anything else that you will like to share?

Thank participant and give gift card
